# Dental trainees reflect more critically on negative experiences: portfolio analysis using a pragmatic approach and a rubric in Japan

**DOI:** 10.1186/s12909-018-1405-x

**Published:** 2018-12-04

**Authors:** Taiji Obayashi, Takayuki Oto, Yukiko Nagatani, Norihiro Taguchi, Hiroyuki Kawaguchi, Tetsuji Ogawa

**Affiliations:** 1 Department of Advanced General Dentistry, Hiroshima University Hospital, 1-2-3 Kasumi Minami, Hiroshima, Hiroshima 734-8551 Japan; 2 Department of General Dental Practices, Kagoshima University Hospital, 8-35-1 Sakuragaoka, Kagoshima, Kagoshima 890-8544 Japan; 3 Dental Hygiene Section, Clinical Support Department, Hiroshima University Hospital, 1-2-3 Kasumi Minami, Hiroshima, Hiroshima 734-8551 Japan; 4 Department of Dental Education, Graduate School of Medical and Dental Sciences, Kagoshima University, 8-35-1 Sakuragaoka, Kagoshima, Kagoshima 890-8544 Japan; 5 Department of Advanced General Dentistry, Hiroshima University Hospital, 1-2-3 Kasumi Minami, Hiroshima, Hiroshima 734-8551 Japan; 6 Graduate School of Letters, Hiroshima University, 1-2-3 Kagamiyama, Higashi-Hiroshima, Hiroshima, 739-8522 Japan

**Keywords:** Reflection, Dental trainee, Dental education in Japan

## Abstract

**Background:**

Dental trainees have various clinical experiences during their internships and they grow by experiencing success and failure. When looking back on an event, it is not apparent which experiences result in more critical reflection. Therefore, we qualitatively analyzed the portfolios of dental trainees using Significant Event Analysis to investigate their depth of reflection.

**Methods:**

We asked all Hiroshima University Hospital dental trainees who completed a 1-year training program between 2010 and 2016 to describe their most memorable event from the past year. We coded the text using a qualitative analysis method. Then, we classified the responses as positive or negative events. We evaluated the depth of reflection following a pragmatic approach to categorizing reflective material (Sandars method) and a reflective ability rubric and user guide (O’Sullivan method) and compared these results. The evaluation was performed separately by two researchers and a good rate of agreement was confirmed by the weighted kappa statistic. Comparison of the depth of reflection was performed by the Mann-Whitney U test.

**Results:**

The assessments of the positive event group and negative event group were compared with the respective evaluation criteria of the Sandars and O’Sullivan methods, and reflection was found to be deeper in the negative event group. The Mann-Whitney U test revealed a significant difference (*p* < 0.05) in the median values of the two groups for both methods.

**Conclusions:**

Both positive and negative experiences are important for dental practitioners to grow, but negative experiences are associated with critical reflections. In promoting the growth of training dentists, it is considered important to encourage deep reflections on positive experiences that are likely to be shallow.

## Background

Medical professionals are required to maintain and improve their clinical skills throughout their lives through acts of continual self-improvement. Dental trainees, who have taken their first steps towards their lifelong journey of training as a member of the clinical world, build upon their past studies (education) in undergraduate education to acquire clinical skills through experiences in the clinical setting and practice continual training (learning) based on the Adult Learning Theory. In the undergraduate education that dental trainees experience, elements of preclinical education are stronger than elements of clinical training. Most have received education from a cognitive learning viewpoint [1] focusing on the learner’s intrinsic information processing process to memorize with class lessons primarily consisting of lectures, and some have been educated from a behavioral learning viewpoint [2] that focuses on active learning that promotes behavioral changes [3]. However, it must not be forgotten that the learning (training) that these dental trainees will undergo through clinical experiences in the lifelong journey of training will mainly consist of the constructivist learning viewpoint [4, 5] of learning through reflection amidst interactions with society (experience pertaining to skills and encounters) [6–8] and the legitimate peripheral participation learning viewpoint [9, 10] of learning through collaboration with others as a member of the clinical setting, rather than the aforementioned cognitive and behavioral learning viewpoint [11]. The legitimate peripheral participation learning viewpoint is a learning viewpoint in which novices who at first can be positioned only in the periphery in workplaces and other social settings come to participate appropriately through learning from senior members and other means and come to acquire a position as a full member of that community.

As a reflective practitioner, medical professionals require the ability to reflect in order to maintain continual self-improvement [12, 13]. Knowledge accumulation and the cognitive placement of meaning (constructivist learning viewpoint) through reflection after action promotes reflection for the next action, and this leads to the self-improvement for acquiring, maintaining, and improving clinical skills [14, 15]. This is the continuation of reflective practice. By undergoing a specific experience and reflectively observing this experience, one makes a generalization within him/herself (“generalization” here refers to meticulously creating “my theory” that is understood only by oneself rather than a concept that is generally understood). “My theory” refers to clinical pearls, techniques, one’s own standards and similar factors. This “my theory” is put into practice and the individual undergoes a new experience and subsequently reflects on this experience, and the cycle repeats. Many unexpected events are encountered in clinical settings, and there are things that an individual cannot deal with using his or her present “my theory.” However, a person cannot run away from a situation simply because it is unexpected. Doctors mobilize their accumulated knowledge and experiences and attempt to somehow get through the situation. The series of reflections performed to do this is called “reflection in action.” Looking back on the situation after it has ended is called “reflection on action,” and a new “my theory” is derived from that reflection. The crucial point is how the experience was interpreted and what kind of information was gained through the experience, rather than the experience itself [16]. In other words, even if multiple people undergo the exact same experience, the learning and subsequent actions differ if the interpretation of the experience differs [17]. As described, experience and learning are concepts that are linked closely and cannot be separated [18], and reflection plays a key role within this process. On the other hand, Moon, who developed a method to analyze and evaluate the depth of reflection from the level and content of critical thinking, reported that critical reflections are closely tied to the acquisition and fostering of clinical skills [19]. Critical reflection as defined by Moon is demonstrating “an awareness that action and events are not only located within and explicable by multiple perspectives, but are located in and influenced by multiple historical and socio-political contexts.” This is strongly associated with expanding the range of and enriching “my theory” and the depth of reflection.

The importance of reflection has been increasing in medical education, and Sandars stated that reflection is an essential aspect of medical education [20]. Onishi et al. proposed a method from a learning strategy viewpoint to promote reflection by introducing Significant Event Analysis (SEA) [21]. With regard to reflection in medical education, prior studies have shown that that Reflective ability scores are higher in students who are provided critical reflection guidelines in advance [22], online reflection writing accompanying feedback improves resident learning [23], and feedback from close relatives and non-medical professionals has positive effects on reflection in medical students’ portfolios [24]. Furthermore, students who exhibit unprofessional behavior have low reflective ability [25].

The training environment of dental trainees affects reflective ability scores [26], and reflective practice interventions in medical care have huge advantages for individual learners, such as making relationships favorable with instructors [27]. Therefore, the Hiroshima University Hospital [28] uses a portfolio for structural reflection as a systematic training tool for postgraduate clinical training for dentists.

In this study, to develop an educational approach and assessment index of critical and deep reflection abilities, we investigated the aspects of reflection in dental trainees who took their first steps towards becoming a medical professional using a portfolio (SEA).

## Methods

Three-hundred and thirty-three dental trainees at Hiroshima University Hospital from FY2010 to FY2016 were asked at the end of the 1-year training (immediately following the graduation ceremony) to reflect on the past year and describe the most memorable event. An A4-size paper was distributed with the question “What was the most memorable event from this past year? Please describe it in your own words below. You can choose to write about anything. There is no word limit. Please describe the event so that it is easy for us to understand why you specifically chose to write about this event.” Although this was conducted after graduation, the trainees were reassured verbally that the questionnaire response would not affect their training evaluation at all and will not be used for purposes other than research. The questionnaire was collected on the same day. The 7 years of data were coded using Steps for Coding and Theorization [29, 30], and concepts were categorized based on the obtained themes and constructs, storyline, and theoretical descriptions. Furthermore, these were categorized into positive events (PE group), negative events (NE group), and neutral events. PE corresponded to descriptions of a happy event, and NE corresponded to a sad or tough event.

The evaluation of a portfolio containing qualitative data requires a different approach than traditional quantitative methods, and the use of rubrics is known to be effective [31]. The depth of reflection was evaluated with the Sandars method [20] and the O’Sullivan method [32]. The former classifies the depth of reflection into six categories (Grade A through F) and is scored in one-point increments from 0 points for Grade F with the shallowest reflection to 5 points for Grade A with the deepest reflection. For the latter classification, the original point system (0 points for the shallowest reflection and 6 points for the deepest reflection) was used. The Sandars method is a modified version of Moon’s four-level assessment [19] and is evaluated on six levels for enhanced practicality (Table 1). The criteria are more specific in the modified version than in Moon’s assessment and are easier to use. On the other hand, the modified version has features in which descriptions involving decisions are scored high even if personal interpretation dictates the decision. O’Sullivan’s method uses a seven-level assessment and is an amended version of the scoring schema that was used at the Centre for Medical Education at the University of Dundee for evaluating reflection of a portfolio (Table 2). The rubric, which specifies the scoring criteria, detailed criteria, and example sentences for each level, was created based on multiple psychometric studies. A study on reflection that used this rubric has been reported [23], demonstrating its reliability and validity. The scoring criteria are easy to understand. Specifically, to attain Level 4 or greater, outside evidence is required. For Level 5, descriptions of a past experience are necessary in addition to meeting Level 4 criteria. Level 6 must meet the criteria for Level 5 and also describe plans for the future. Naturally, due to the requirement of outside evidence, the assessment would remain at Level 3 if it only includes personal evaluations, even if past experiences are referenced and the cause of the event and solutions are described in depth. In this study, both methods were used with consideration of the features of each evaluation method and to verify the assessment from various perspectives.

**Table 1:** Scores of Reflection Measured by the Sandars method

Score	Scoring Guidelines
0 - (Grade F)	Describing an event – poor description of an event.
1 - (Grade E)	Describing an event – repeating the details of an event without offering any interpretation.
2 - (Grade D)	Describing an event – recognising that something is important but not explaining why.
3 - (Grade C)	Describing an event – recognising how it affects your feelings, attitudes and beliefs and/or questioning what has been learnt and comparing it to previous experience.
4 - (Grade B)	Involves judgement – what went well, or less well and why.
5 - (Grade A)	Experiencing an event(s) has changed, or confirmed, how you experience an event(s). You may wish to change how you respond to similar event(s in the future. You provide an explanation, including references to other literature, eg articles or books.

**Table 2:** Level of Reflection Measured by the O’Sullivan method

Level	Reflection Performance
0	Does not respond to the assignment
1	Describes without reflecting
2	Does not justify lessons learned
3	Provides limited justification of lessons learned
4	Includes evidence of lessons learned
5	Analyzes factors from experience
6	Integrates previous experience with current events and data to inform further action

The evaluation was performed by two researchers, and a satisfactory inter-rater agreement was confirmed with weighted kappa statistics (weighted κ > 0.8). After verifying the homogeneity of dispersion with the Siegel-Tukey test [33], the depth of reflection was compared between the PE group and NE group with the Mann-Whitney U test (two-tailed test). This study was approved by the Hiroshima University Epidemiological Studies Ethics Committee.

## Results

The survey collection rate was 42/43 in FY2010, 52/55 in FY 2011, 51/54 in FY2012, 38/48 in FY2013, 43/49 in FY2014, 48/49 in FY2015, and 34/36 in FY2016. The collection rate was low in FY2013 because seven individuals were absent from the graduation ceremony. Based on qualitative analysis over the 7-year study period, 152 (45.6%) trainees were categorized into the PE group, 72 (21.6%) in the NE group, and 109 (32.7%) in the neutral group. The neutral group gave responses that could not be categorized as either positive events or negative events, and were excluded from the analysis. The results from the Mann-Whitney U test are shown in Table 3.

**Table 3:** Results of the Mann-Whitney U test between the PE group and NE group

	Sandars method							
	2010	2011	2012	2013	2014	2015	2016	Overall
PE group (n)	19	32	29	18	23	20	11	152
NE group (n)	5	16	19	6	12	6	8	72
PE (median)	2	2	2	2	1	1.5	1	2
NE (median)	3	3	3	3	2	2.5	2	2.5
U-value	19	163.5	180	22	52	29.5	14	3063.5
*P*-value	0.0439	0.0354	0.0357	0.033	0.0008	0.0478	0.0037	*p* < 0.0001
	O’Sullivan method							
	2010	2011	2012	2013	2014	2015	2016	Overall
PE group(n)	19	32	29	18	23	20	11	152
NE group (n)	5	16	19	6	12	6	8	72
PE (median)	1	2	2	1	1	1	1	1
NE (median)	3	3	3	3	2	3	2	3
U-value	14	117	135	15	70	27	19.5	2660
*P*-value	0.012	0.002	0.001	0.007	0.012	0.0307	0.013	*p* < 0.001

PE: positive event; NE: negative event; n: number of people, median: median depth of reflection per person. Overall includes data from all 7 years

**Fig. 1:**
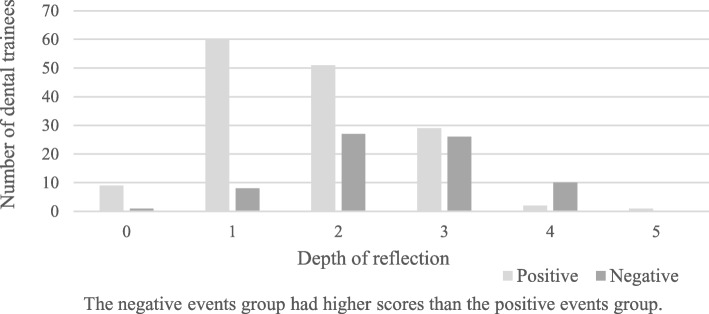
Comparison of positive and negative events (Sandars method)

**Fig. 2:**
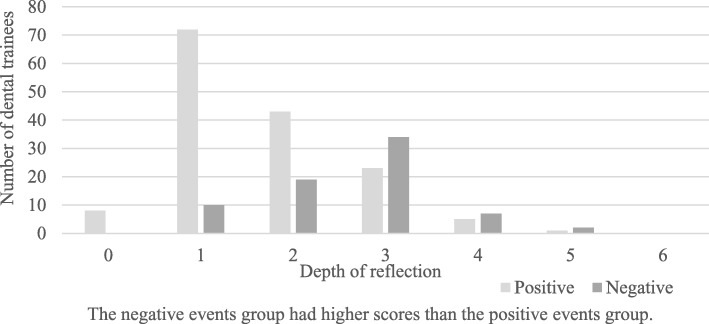
Comparison of positive and negative events (O’Sullivan method)

The PE group and NE group were compared each year and overall (all 7 years) with the two types of evaluation criteria. The results showed that the medians for the PE and NE groups were significantly different in all Mann-Whitney U tests using both the Sandars method and O’Sullivan method. The frequency distributions of the depth of reflection in the PE group and NE group over the 7-year period are shown in Figs. 1 and 2.

Below are examples of an SEA classified in the PE group.

“The first patient I saw in the general internal medicine department was my first opportunity to provide treatment as a dentist, but the patient wanted new partial dentures. …It was the first time I had made partial dentures. As I did this my heart was thumping inside, but I studied with the feeling of “I can do this” and 1 week later we talked about making the dentures. It took some time, but after they were made the patient said “They’re better than the ones I have now!” I remember his name and I remember his face. He left a real impression. At that time I felt for the first time that I had become a dentist. I was happy and that night had some drinks. Why write that? Simply because I was happy. That experience gave me confidence. I thought that hard work would be rewarded and the feeling would be conveyed.”

This is a typical example classified in the PE group. Events where things went well are described, but there is no comparison with past experiences and there is no description of why things went well.

Now, here is an example of an SEA classified in the NE group.

“I made a perforation when performing a lower right molar pulpectomy. I had a certain amount of experience with pulpectomy in the past, and so I was a little overconfident in my ability to perform it. There were many reasons for the perforation, including that an isolated tooth was leaning quite mesially to the tooth axis, that there was significant caries in the cervical portion of the tooth and it was difficult to see the relationship with the floor of the pulp chamber, that I did not fully grasp the anatomical characteristics of the molars, that the target line could not be completely set when looking at X-ray images and inside the oral cavity, and that I did not ask the advising doctor to confirm the procedure midway, but it is an event that has really stuck with me. I wanted to study so that it would never happen again.”

This individual reflects from the point of view that there were multiple causes for this failure. This is a typical example of the deep reflection seen in the NE group.

Examples of SEA data concept classification results are shown below.

PE Group.

“Patient satisfaction leads to professional descriptions”.

“Self-improvement is rewarded in clinical practice”.

“Trust from a respected advising doctor model”.

“Joy of working together with colleagues who have diverse backgrounds,”

NE Group.

“Regret arising from underdeveloped clinical skills”.

“Fate of medical care providers is to be in a position to know life expectancies”.

“Advising doctor-resident relationship could not be built,”

## Discussion

This study analyzed a questionnaire that was distributed immediately after a training completion ceremony. The questionnaire asked certified trainees to reflect on the past year of training and describe a memorable event. In all 7 years, more trainees described a PE than a NE, and this may have been due to their awareness of starting a new life in a new environment from the new fiscal year. For example, it has been reported previously in a psychological study that an individual’s successful experience becomes a source of motivation to continue actions [34] and it has also been shown that employment is an important category as a turning point in young adulthood for the future [35].

Comparing the depth of reflection between the PE group and NE group, it was revealed that the reflection was significantly deeper in the NE group than PE group in all years using either evaluation method. This finding corroborates the hypothesis that dental trainees may reflect on a negative event more deeply than a positive event [36]. This hypothesis was obtained in an analysis of questionnaires from dental trainees at the authors’ hospital in 2011. The questionnaire clarified aspects of reflection at a midpoint in training and showed that while equal numbers of people described negative events and positive events, reflection was significantly deeper in the NE group than in the PE group.

Here, we will discuss the reflections on PE and NE experiences. Previous studies on reflection in the behavior sciences and psychology fields primarily pertained to NE experiences because reflection on successful experiences is not effective for self-growth [37]. For example, Sitkin stated the importance of “strategic small losses,” a design for loss and failure promotion in an organization [38]. However, several studies on the importance of reflecting on a PE experience emerged this century. Ellis and Davidi asked Israeli army soldiers to reflect on training tasks [39]. Half of the soldiers were asked to reflect on failures while the other half were asked to reflect on both successes and failures. When the performance improvement was compared between the two groups, the latter group had better training task performance than the former group. Based on these findings, Tsumagari stated that “to improve human behavior and performance, it is indispensable not only to analyze the cause of failure, but also to clarify the factors for success and to acquire the principles of success” [34]. Moreover, it has been reported that the mental stability gained through analyzing a successful experience facilitates and eases the analysis of failed experiences [40], indicating that not only the reflection of failed experience, but also the reflection of successful experience, plays an essential role in personal growth.

The results of these previous studies and those of the present study were compared. With regard to the importance of NE experiences, the finding of the present study that reflection was deeper with NE experiences than with PE experiences supports results of earlier studies in the twentieth century of the importance of NE experiences. However, considering the results of studies in recent years that successful experiences play an important background role when people reflect back on experiences of failure, attention needs to be directed to the result of this study that reflection in PE experiences is shallow. Analysis of shallow successful experiences is, put simply, typically along the lines of “I don’t really know why things went well, but I’m glad they did.” However, the true mental stability obtained from success may be based less on the simple joy of succeeding and more on the joy obtained from reflection with analysis of why things went well and what was different from the past that led to success, which allows experiences of failure to be analyzed while maintaining a sense of confidence.

The frequency of PE and NE was found to be a PE:NE ratio of about 2:1, but past studies have also shown the possibility of effects from the mental stability produced by the analyses of successful experiences [40]. That is, when describing an event that remained in one’s mind the most over a year, it seems that most people prioritized the mental stability obtained from describing PE experiences rather than recalling a bitter experience by analyzing and writing about an NE experience.

Two methods were used in this study, but as mentioned in the Methods section, each of these evaluation methods has its own characteristics. Even so, the results were the same. This shows that in evaluations with either set of characteristics, reflections on NE experiences are shown to be deeper than those on PE experiences. This is thought to produce added value in terms of increasing the reliability of the results of this study.

This study showed that the reflections of dental trainees are deeper in NE experiences than in PE experiences, indicating the importance of encouraging deep reflections of PE experiences, which tend to be reflected in a shallow manner, for the promotion of growth in dental trainees. The reconstruction of knowledge requires self-examination of the knowledge and experience that an individual already possesses, but this process can become more profound by interacting with others [41]. Thus, the facilitation of structural reflections through conversations between dental trainees or between dental trainees and supervisors as well as coaching skills that are used in the business field are considered to be effective. Coaching is the process of equipping the other person with the knowledge and skills needed to achieve goals and encouraging behaviors in working toward those goals through repeated conversations. For many of those skills, the process of developing and working toward goals oneself is supported and stimulated, and these things are thought to have much in common with encouraging reflection [42].

Both PE and NE experiences are essential in the growth process and it is important to promote deeper and more critical reflection on PE experiences that are easy to gloss over to foster the clinical abilities of dental trainees. We would like to propose using a showcase portfolio (a portfolio consisting of best work chosen by trainees themselves) [43] in the final stage of an initial clinical training program, in which trainees present one case each of PE and NE corresponding to the “most outstanding result” and “most disappointing result” and the entire group discusses why each case turned out as it did. Prior to the start of training, the rubric should be disclosed and explained, and having students write their portfolios may help to deepen their reflection. If it is difficult to disclose and explain the rubric, simply giving advice such as “write your descriptions while comparing with past experiences” is also acceptable, since even that can be training that encourages deep reflection. We believe that the results of this study can help to develop educational methods and evaluation indicators of critical refinement ability in the future. Previous studies have reported that reflections on the portfolio evaluations of students who exhibited unprofessional behavior are shallower than those of other students [26], and we think that the depth of reflections are related to outcomes.

### Limitations of this study

To clarify the principles of the aspects of reflection in clinical dental trainees at Hiroshima University Hospital, this study investigated the depth of reflection of PE experiences and NE experiences in a limited study population without accounting for various factors.

In general, research methods are roughly divided into quantitative and qualitative studies. In this study, we used SEA, a quantitative method of scoring qualitative data, for comparisons. However, the categorization of PE and NE were completed qualitatively. Because the question in this study relates to the elucidation of the essence and basic principles of reflection, it is difficult and unsuitable to conduct the analysis with a quantitative method alone, and a qualitative method is appropriate from an interpretivist perspective [44]. Therefore, the generalizability is limited, and while the directionality indicated by this study may not be applicable in general, it was suggested based on the principles of analogy [45] that similar occurrences as the present study findings may be taking place in institutions with a comparable environment.

The data used in this study were obtained immediately after the training completion ceremony, and therefore, the possibility that the mood at that time affected the content of the responses cannot be ruled out. However, the graduation ceremony at our hospital is very businesslike and in addition to the data for this study, other administrative documents were also completed, and so the effects are thought to be very limited.

## Conclusions

We examined the portfolios (SEA) of dental trainees at Hiroshima University Hospital from FY2010 to FY2016 and found that dental trainees reflected more critically on NE experiences than PE experiences.

## Abbreviations

NE: Negative event
PE: Positive event
SEA: Significant Event Analysis
